# The influence of perfusion solution on renal graft viability assessment

**DOI:** 10.1186/2047-1440-1-18

**Published:** 2012-10-18

**Authors:** Colin H Wilson, Hugh Wyrley-Birch, Dhakshinarmoorthy Vijayanand, Anabelle Leea, Noel M Carter, Malcolm Haswell, Anne C Cunningham, David Talbot

**Affiliations:** 1 Applied Immunobiology Group, University of Sunderland, Sunderland, UK; 2 The Institute of Transplantation, The Freeman Hospital, Newcastle-upon-Tyne, UK

## Abstract

**Background:**

Kidneys from donors after cardiac or circulatory death are exposed to extended periods of both warm ischemia and intra-arterial cooling before organ recovery. Marshall’s hypertonic citrate (HOC) and Bretschneider’s histidine-tryptophan-ketoglutarate (HTK) preservation solutions are cheap, low viscosity preservation solutions used clinically for organ flushing. The aim of the present study was to evaluate the effects of these two solutions both on parameters used in clinical practice to assess organ viability prior to transplantation and histological evidence of ischemic injury after reperfusion.

**Methods:**

Rodent kidneys were exposed to post-mortem warm ischemia, extended intra-arterial cooling (IAC) (up to 2 h) with preservation solution and reperfusion with either Krebs-Hensleit or whole blood in a transplant model. Control kidneys were either reperfused directly after retrieval or stored in 0.9% saline. Biochemical, immunological and histological parameters were assessed using glutathione-*S*-transferase (GST) enzymatic assays, polymerase chain reaction and mitochondrial electron microscopy respectively. Vascular function was assessed by supplementing the Krebs-Hensleit perfusion solution with phenylephrine to stimulate smooth muscle contraction followed by acetylcholine to trigger endothelial dependent relaxation.

**Results:**

When compared with kidneys reperfused directly post mortem, 2 h of IAC significantly reduced smooth muscle contractile function, endothelial function and upregulated vascular cellular adhesion molecule type 1 (VCAM-1) independent of the preservation solution. However, GST release, vascular resistance, weight gain and histological mitochondrial injury were dependent on the preservation solution used.

**Conclusions:**

We conclude that initial machine perfusion viability tests, including ischemic vascular resistance and GST, are dependent on the perfusion solution used during *in situ* cooling. HTK-perfused kidneys will be heavier, have higher GST readings and yet reduced mitochondrial ischemic injury when compared with HOC-perfused kidneys. Clinicians should be aware of this when deciding which kidneys to transplant or discard.

## Background

Donors after circulatory death (DCD) are increasingly being utilized by transplant centers in response to rising numbers of patients on organ waiting lists [1]. These donors are declared dead on the basis of cardiorespiratory parameters, rather than brain stem function, and there is therefore a period (10 to 40 minutes) of asystole and warm ischemia before organ retrieval. In the UK the number of DCDs increased over 70% between 2007 and 2011 [2].

The majority of these UK centers use controlled donors: patients declared dead in critical care units and rushed to the operating theatre for organ recovery using a standard aortic cannula to cool the abdominal organs [3]. Uncontrolled DCDs, where a patient is declared dead outside or shortly after reaching hospital, in the majority of centers, are cooled using a double balloon triple lumen (DBTL) catheter system capable of isolating the abdominal circulation via the femoral artery prior to organ retrieval. In both situations there is an extended period of warm ischemia (10 to 30 minutes) and a further extended period of intra-arterial cooling (IAC) (up to 2 h for uncontrolled) whilst the donor is transferred to theatre and/or the organs mobilized for topical cooling and recovery [4,5]. A critical feature of this period is that high flow IAC must be maintained to cool the renal parenchyma or the kidneys are ‘backwashed’ with warm blood from the thorax. As a result large quantities of preservation solution (up to 25 l) can be required in the uncontrolled situation [5].

Viscous solutions like University of Wisconsin (UW) solution have traditionally been too costly for this purpose, and Marshall’s hypertonic citrate (HOC) [6] or Bretschneider’s histidine-tryptophan-ketoglutarate (HTK) [4,5] have been preferred, whilst sanguineous perfusion techniques using extracorporeal membrane oxygenation requires dedicated specialist teams and remains largely experimental [7,8].

Currently the risk that a kidney will never function (primary non-function) remains higher with DCDs than with either live or brain stem dead donors (circa 5% vs 1%) and [9,10] a number of centers, including our own, have a program of viability testing to identify severely damaged organs using hypothermic machine perfusion before implantation [9,11]. Perfusion criteria predicting early graft failure include high vascular resistance (pressure divided by flow taking into account the weight of the kidney; pressure flow index (PFI)) and high perfusate glutathione-*S*-transferase levels (GST) [4,11,12].

In previous work we have highlighted the differences in endothelial preservation using different perfusion solutions and have reported clinical data comparing kidneys perfused in HTK and HOC [13,14]. This set of experiments was designed to compare ‘whole organ’ kidney perfusion preservation with HTK and HOC, with particular emphasis on warm ischemia and vascular function, in order to understand the implications of our previous clinical and experimental observations.

## Methods

### IAC and estimation of weight gain

**Figure 1:**
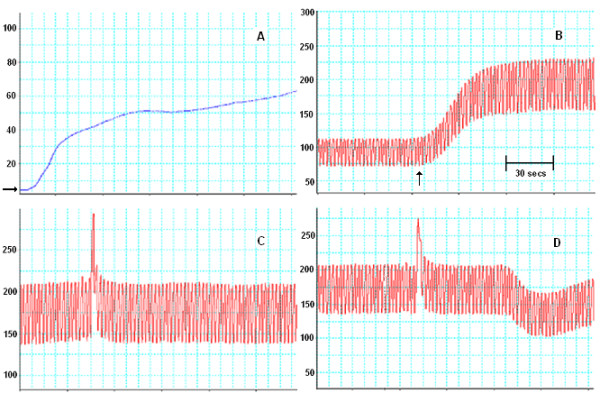
**Representative recorded perfusion traces of a single kidney. **(**A**) Pressure during intra-arterial cooling (IAC) with histidine-tryptophan-ketoglutarate (HTK) (blue); (**B**) after pulsatile reperfusion with Krebs Hensleit (red) reperfusion media. The arrow represents start of supplementation with phenylephrine and a rapid rise in mean pressures to around 200 mmHg. In (**C**) a 200 μl vehicle bolus is given with no change in mean perfusion pressure. However, in (**D**) 200 μl acetycholine stimulates endothelial dependent relaxation approximately 45 s later. Y-axis, mmHg; X-axis time (s)

Male Wistar rats (250 to 350 g) were killed by cranial stunning followed by cervical dislocation in accordance with Schedule 1 of the UK Animals (Scientific Procedures) Act 1986. Immediate laparotomy was then performed and the left kidney reflected to expose the renal artery into which a 20 G cannula was secured. The kidney was then dissected free and weighed with cannula in place. IAC was initiated after a total of 30 minutes post-mortem warm ischemia with a 500-μl flush of streptokinase (144 IU Streptase®; Aventis Behring GmbH, Marburg, Germany) via the cannula [15]. Preservation solution (HTK or HOC) was then injected at a fixed flow rate (25 ml/h) having passed through a cooling circuit (6 to 10°C). Pressure was monitored throughout the experiment using a Power Lab® 8e (AD Instruments; New South Wales, Australia) transducer and the results recorded using Chart v.5 (AD Instruments) software (Figure 1A). After 2 h the kidney was reweighed and transferred to the warm circuit for reperfusion (HTK and HOC, n = 8). Weight gain was calculated by subtracting the weight of the cannula from both readings and expressing the increase as a percentage. Negative control kidneys (n = 9) underwent the same protocol using normal saline as the preservation solution. Positive control kidneys (Control, n = 9) were flushed directly after cannulation with streptokinase and reperfused on the warm circuit; an ischemic period of between 12 and 15 minutes. In further experiments kidneys were stored in the same preservation solution (n = 6) for 20 h and then reperfused with whole blood prior to mitochondrial analysis (see below).

### Reperfusion (‘warm’) circuit

The warm circuit consisted of a rotor pump (Watson-Marlow; Falmouth, UK) capable of delivering pulsatile flow rates between 1 and 10 ml/min (Figure 1B-D). The perfusate (Krebs-Hensleit solution) was made to previously published specifications and filter sterilized before use [16]. Perfusate was pumped from a warmed (37°C), oxygenated reservoir via a bubble trap and secondary warming coil into the kidney. Perfusate effluent was drained from round the kidney by a separate circuit and either stored for analysis or discarded. Preliminary experiments established that the system maintained both a cannula tip and perirenal temperature in the range of 37 ± 0.5°C. For the first 30 minutes of reperfusion the flow was adjusted to maintain a mean arterial pressure of approx. 90 mmHg. At 30 minutes post reperfusion the flow was fixed and phenylephrine (see below) added to the perfusate (Figure 1B).

### Measurement of vascular resistance (VR)

During both IAC and the second 30 minutes of reperfusion, mean arterial pressure was used as a surrogate for VR (fixed flow). VR during the first 30 minutes of reperfusion was calculated using the following formula:

$$\text{VR}=\frac{\text{mean}\;\text{arterial}\;\text{pressure}\;\left(mmHg\right)}{\left(\text{Flow}\;\left(ml/\text{min}\right)/\text{Pre}z-\text{perfusion}\;\text{kidney}\;\text{weight}\left(g\right)\right)}$$

### Assessment of vascular function

Smooth muscle function was assessed as the maximal contractile response to the perfusate supplemented with the α-adrenergic agonist phenylephrine (10 μM) and reported as increase in VR (Figure 1B). At 45 minutes post reperfusion three boluses of the perfusate vehicle (200 μl) were given via an injection port in the circuit to ensure there was no agonist contamination (Figure 1C) and then four similar volume boluses of acetylcholine (2 μM, 20 μM, 200 μM, 2,000 μM) injected at 3-minute intervals (Figure 1D) to stimulate endothelial dependent relaxation [16]. A final bolus of papaverine (10 mM) at 1-h post reperfusion elicited endothelial independent relaxation. This is reported as a percentage of the initial phenylephrine induced contraction and endothelial independent relaxation calculated, for each acetylcholine bolus, as a percentage of the papaverine relaxation.

### GST activity

Samples of perfusate effluent were taken each hour during IAC and every 15 minutes during reperfusion and snap frozen for GST enzyme activity analysis, which is a marker of renal tubular cell damage. This was performed using a previously published spectrophotometric method on an automated analyzer [17].

### Real-time polymerase chain reaction (PCR)

After the end of reperfusion (1 h) a 5 × 3 mm section was removed from the upper pole of each kidney and stored in RNAlater® (Ambion, Texas, USA). RNA extraction was performed with an initial homogenization in 1 ml of phenol:guanidine thiocyanate solution (RNAzol B®; IsoTex Diagnostics, Texas, USA) combined with chloroform. Subsequent isopropanol and ethanol washes produced a nucleic acid/protein pellet which was further purified using phenol:chloroform and centrifuged with Phase Lock Gels® (VWR International, Leicester, UK). Total RNA content and integrity were assessed using spectrophotometry (absorbance 260 nm) and gel electrophoresis before cDNA synthesis with an MMLV reverse transcriptase (Bioline, London, UK). At this stage the positive control kidney cDNA was pooled and aliquots used in subsequent amplifications as a consistent reference. Validated exon-spanning TaqMan® primers (B-actin Rn 00667869; intercellular adhesion molecule type 1 (ICAM-1) Rn 00564227; vascular cellular adhesion molecule type 1 (VCAM-1) Rn 00563627; RANTES (for ‘regulated upon activation, normal T cell expressed and secreted’) Rn 00579590 ml; Applied Biosystems, CA, USA) were used during amplification on a real-time quantitative thermal cycler (RG-3000; Corbett Research, Sydney, Australia). PCR products were quantified using the 2^-ΔΔCT^ method [18], normalizing to B actin, and the results for each experimental replicate expressed as fold changes relative to control.

### Whole blood ‘buddy’ reperfusion

After IAC a number of kidneys were recovered for reperfusion using a ‘recipient’ animal after 20 h cold ischemia. Each kidney recovered was reperfused by a separate, male Wistar rat of similar size to the donor. In brief, the recipient animal was anaesthetized by gas induction (isoflurane) and maintained with intraperitoneal and intravenous boluses of Hypnorm® (Janssen, Oxford, UK; fentanyl citrate 0.15 mg/ml; fluanisone 10 mg/ml) and midazolam. The left femoral vessels were exposed, controlled (bulldog clips) and cannulated with heparin coated, PTFE cannulae (Instech Laboratories, Plymouth, USA; 3 F in artery, 3.5 F in vein). Cannula patency was maintained by heparinization of the recipient (single bolus of 100 IU). The renal vessels were then secured using surgical ties and the vessel clips released to initiate reperfusion. After 4 h, reperfusion was terminated by dividing the renal vessels at the hilum and euthanizing the recipient. The recipient’s native left kidney was recovered immediately post mortem for use as a negative control (minimal ischemic injury).

### Mitochondrial injury

**Figure 2:**
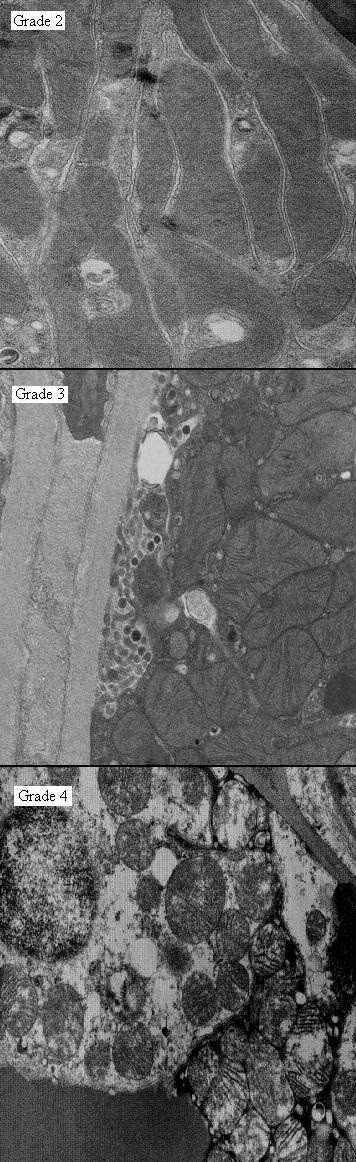
**Trump grades 2 to 4 of mitochondrial injury.** Well-preserved mitochondria, showing elongated, orthodox conformation (injury grade 2). Mitochondria showing injury with condensation of their inner compartments, increased density of the matrices and expansion of intracristal spaces (grade 3). Mitochondria showing maximal, reversible injury with markedly condensed matrices and markedly expanded intracristal spaces. The inner compartments are expanded and those with flocculent densities may have irreversible injury (injury grade 4)

Renal tissue was prepared for electron microscopy using a previously described fixation method [14]. For each kidney sample, 40 high powered fields were selected at random and the mitochondria in each field were assigned an injury grade by a single observer blinded to the identity of the sample (MH). The Trump scale for mitochondrial injury was used to assess viability [19]. This identifies seven grades of renal tubular injury, on a linear scale, ranging between potentially viable tissue (1 to 4) and non-viable tissue (5 to 7) (Figure 2).

### Statistical analysis

Unless otherwise specified parametric data is presented as mean ± standard error of the mean (SEM) and compared using analysis of variance (ANOVA) with the Bonferroni *post hoc* test. Non-parametric data was identified using the Kolmogorov-Smirnov test, and presented as the median and range. Between-group comparisons were made with the Kruskal-Wallis test or Mann-Whitney U test depending on the number of groups. Error bars represent the standard error of the mean and range for normally distributed data and skewed data respectively. Statistical significance was interpreted as a *P* value < 0.05 for the two-sided hypothesis. All calculations and graphical representations were performed using Prism V.4 (Graphpad Software Inc.; San Diego, California, USA).

## Results

### Vascular resistance during intra-arterial cooling

**Figure 3:**
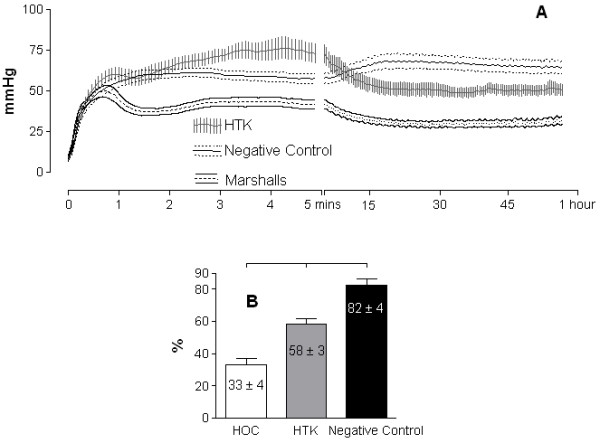
**(A) Intra-arterial cooling (IAC) perfusion profiles with histidine-tryptophan-ketoglutarate (HTK) (n = 8), Marshall’s hypertonic citrate (HOC) (n = 8) and normal saline (negative control n = 9).** Lines and bars represent mean ± SEM. Data abbreviated at 1 h (analysis of variance (ANOVA) with Bonferroni *P* < 0.001). (**B**) Weight gain, same data sets, after IAC; mean ± SEM (t test *P* < 0.01)

There were no significant differences in the kidney weights between the experimental groups (1.29 ± 0.01 g, range 1.0 to 1.45) (Figure 3A). All the kidneys had a similar perfusion profile over the first minute of IAC, reaching a pressure of around 50 mmHg within the first minute. After this time point VR varied dependent on the preservation solution used, rising with HTK (maximum 76.7 mmHg at 4 minutes) or normal saline (max. 68 mmHg at 21 minutes) and dropping with Marshall’s HOC (mean plateau 29 mmHg).

### Weight gain during intra-arterial cooling

All the kidneys became edematous during IAC, noticeable macroscopically, although the magnitude of this gain was related to the preservation solution: HOC 33 ± 4% and HTK 58 ± 3% having statistically (*P* < 0.01, ANOVA with Bonferroni) less weight gain than with negative control 82 ± 4% (Figure 3B).

### Vascular function after reperfusion

**Figure 4:**
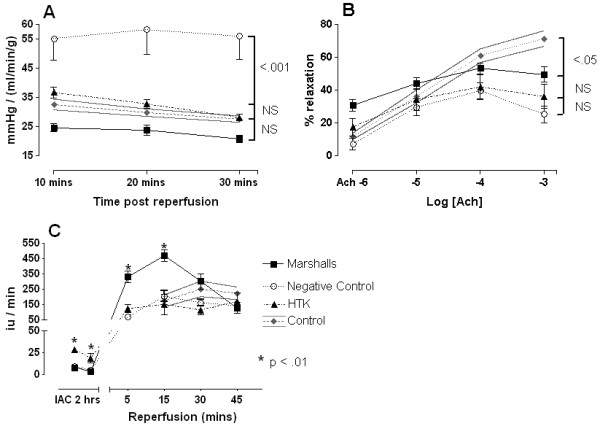
**Reperfusion variables (I). **(**A**) Vascular resistance during reperfusion. (**B**) Endothelial dependent relaxation as a percentage of phenylephrine contraction. (**C**) Glutathione-*S*-transferase (GST) release during intra-arterial cooling (IAC) and reperfusion. Lines and bars represent mean and SEM, *P* values, analysis of variance (ANOVA) with Bonferroni. Histidine-tryptophan-ketoglutarate (HTK) and hypertonic citrate (HOC) (n = 8), negative control (n = 9) NS, non-significant

**Figure 5:**
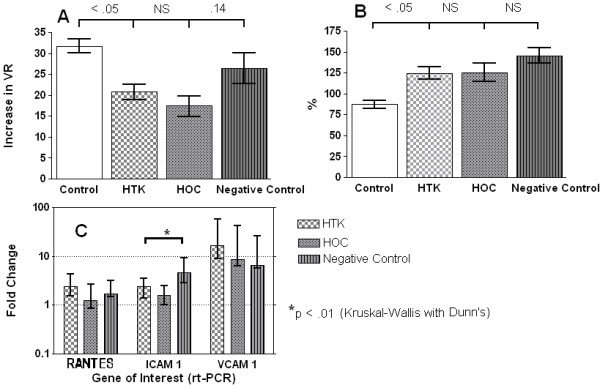
**Reperfusion variables (II). **(**A**) Contractile response to phenylephrine. Bars represent mean ± SEM, *P* values, analysis of variance (ANOVA) with Bonferroni. (**B**) Endothelial independent relaxation in response to papaverine bolus. Percentage of phenylephrine contraction. Bars represent mean ± SEM, *P* values, ANOVA with Bonferroni. (**C**) Immune activation (mRNA) as measured by real-time polymerase chain reaction (PCR) normalized to B actin and expressed as fold change over positive control. Bars represent median and range. Histidine-tryptophan-ketoglutarate (HTK) and hypertonic citrate (HOC) (n = 8), negative control (n = 9). NS, non-significant

The VR during IAC was reflected in the VR at reperfusion with the negative control kidneys not only having the highest values at all time points (*P* < 0.001), but also showing no indication of improving over time (Figure 4A). In contrast both HTK and HOC had VRs comparable to positive control, which improved within the short period of reperfusion and was accompanied by the washout of erythrocytes before the addition of phenylephrine. The response to this agonist was blunted in the experimental groups (Figure 5A) and endothelial independent relaxation greater when compared with the positive controls (Figure 5B). Endothelial dependent relaxation was reduced in all the experimental groups (*P* < 0.05) and lowest in the negative control group, although there were no significant differences between the preservation solutions (Figure 4B).

### GST, RANTES and adhesion molecules

The GST values highlighted a major difference in renal tubular cell response to ischemia reperfusion injury (IRI) dependent on the preservation solution. The values seen during ischemia were higher in HTK-perfused kidneys than Marshall’s, but during reperfusion this ratio was reversed with almost a threefold difference in GST perfusate readings at 15 minutes. Of the adhesion molecules, ICAM-1, was significantly overexpressed when compared with Marshall’s, but not HTK, and VCAM-1 was the most globally upregulated of the three tested (8-fold to 57-fold). See Figures 4C and 5C.

### Mitochondrial injury

**Figure 6:**
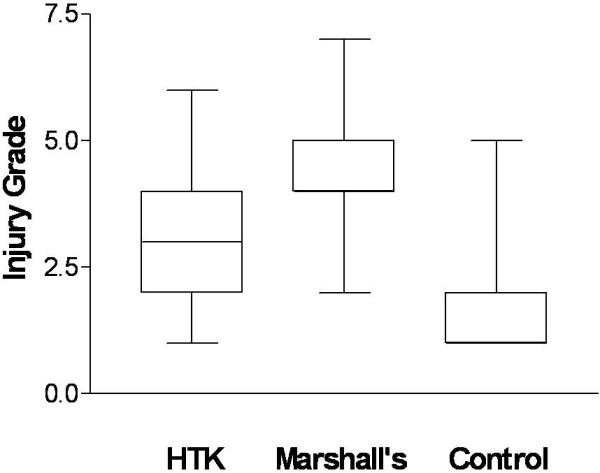
**Mitochondrial injury.** Electron microscopic comparison by blinded observer of mitochondria according to Trump scale of injury. Histidine-tryptophan-ketoglutarate (HTK), Marshall’s hypertonic citrate (HOC) and control (n = 6). Lines and bars represent median and range with interquartile box. Data significant at *P* < 0.0001, Mann-Whitney U test

There were significant differences between kidneys perfused and stored in the two preservation solutions when compared with kidneys recovered post mortem (Figure 6). Kidneys preserved in HTK had a median injury grade of 3 (interquartile range 2 to 4) whereas those preserved in Marshall’s had a grade of 4 (interquartile range 4 to 5) and this difference was statistically significant (*P* < 0.0001).

## Discussion

The vascular system is more than just a conduit for oxygenated blood to peripheral tissues. The interaction between vascular smooth muscle cells, endothelium and luminal contents regulates vascular tone and barrier function by cells responding to and producing vasoactive mediators, adhesion molecules and chemokines. In these experiments we have shown that warm ischemia and intra-arterial cooling have variable effects on organ vascular function and mitochondrial integrity some of which are dependent and some of which are independent of the preservation solution.

The vascular resistance during donor perfusion was strikingly different between HTK and HOC when compared with normal saline. In further experiments, not reported here, we changed the osmolarity of the three solutions with mannitol and showed that this manipulation was responsible for the majority of the difference in perfusion pressures (unpublished data). This was also mirrored in the weight gain: edema being directly correlated with osmolarity and flow. In a porcine study using controlled pressure perfusion, weight gain with HOC was 50%, suggesting that the overall volume and flow in this study were not excessive [20].

However, the differences in endothelial dependent relaxation between HTK and HOC in response to acetylcholine were less striking than our previous experiments in rodent aorta had suggested: this may reflect the shorter period of ischemia or a true tissue specific difference. We did try and record acetylcholine responses after long periods of cold storage, but the results were confounded by very low response rates in all preservation media.

Endothelial independent relaxation was numerically greater than the positive control (see Figure 5C), but this probably represents a higher level of basal vascular tone pre-phenylephrine (these figures exceed a 100% of the induced vasoconstriction) rather than actual enhanced responsiveness. Markers of immune activation (Figure 5C) were also upregulated relatively independently of preservation solution and quantitatively were greatest for the vascular endothelial adhesion molecule VCAM-1 which can be overexpressed on renal tubular as well as vascular endothelial cells [21,22].

Choice of perfusion solution did have a significant bearing on the measured release of the GST viability enzyme (Figure 4C); with a threefold increase in the amount of tubular cell death in the HOC perfusion group when compared with HTK, negative and positive controls. We do not believe that saline provides better protection for the renal tubular cell than either HTK or HOC; a more plausible explanation would be that lack of tissue perfusion prevented a complete extrusion of the released GST from the negative control treated kidneys in the short time frame. The contrast between GST release during ischemia and reperfusion of kidneys perfused with HTK and HOC suggests a ‘paradox’ type injury. We speculate that the greater pH buffering capability of HTK makes the kidney confusingly susceptible to cellular damage during ischemia [23] but relatively protected during reperfusion. Conversely, with HOC treated kidneys, the greater acidosis is initially protective, [24] but reperfusion and resumption of aerobic metabolism triggers proteolytic enzyme activity and exaggerated cell death [25]. Hence, a minor increase in tubular damage seen with HTK during ischemia, precedes a much larger reperfusion ‘hit’ with HOC. This would concur with other studies documenting the relative lack of protection conferred by HOC on reperfusion injury after warm ischemic injury [14,26,27]. Of course, most DCD kidneys are transplanted after *ex vivo* preservation with either UW static storage solution or after a period of hypothermic perfusion preservation. It is unclear whether this period effectively abrogates the differences between HTK and HOC or whether there are specific ‘interactions’ between different combinations of solutions, which are either beneficial or harmful. In common with other units we would not advocate storage of DCD kidneys in either HTK or HOC [28].

## Conclusions

The most relevant and immediate clinical deductions from these experiments are the implications for viability testing of kidneys. All other factors being equal, centers using HTK for IAC could reasonably expect the retrieved kidneys to be larger, have a higher initial resistance and ischemic GST values than kidneys exposed to warm ischemia and HOC for a similar period of time. Indeed, in our own clinical transplant program we have noticed that this is the case and it was these observations that stimulated this research (see introduction [13]). The mitochondrial injury studies have clear viability implications for perfusing and storing kidneys with HOC and we have stopped perfusing or storing DCD kidneys in HOC. Further experimental work is required to examine the interactions between HTK and HOC with *ex vivo* perfusion preservation and the UW type preservation solutions.

## Competing interests

The authors declare that they have no competing interests.

## Authors’ contributions

CW: manuscript, rodent kidney perfusion and concept. HWB: ‘buddy reperfusion’ model and electron microscopy. DV: developed ‘buddy reperfusion’ model with HWB. AL: mitochondrial injury model. NC: PCR and reviewed manuscript. MH: electron microscopy. AC: manuscript review and development of experimental techniques. DT: concept development and guarantor. All authors read and approved the final manuscript.

## References

[B1] Shoskes D Warming to non-heart-beating donors? Am J Transplant 2001 1 305 306 10.1034/j.1600-6143.2001.10402.x 12099371

[B2] NHSBT National Health Service Blood and Transplant Activity Report 2009/2010: Transplant Activity in the UKhttp://www.organdonation.nhs.uk/ukt/statistics/transplant_activity_report/current_activity_reports/ukt/activity_report_2009_10.pdf

[B3] Johnson SR Pavlakis M Khwaja K Karp SJ Curry M Curran CC Monaco AP Hanto DW Intensive care unit extubation does not preclude extrarenal organ recovery from donors after cardiac death Transplantation 2005 80 1244 1250 10.1097/01.TP.0000179643.56257.7F 16314792

[B4] Booster MH Wijnen RM Vroemen JP van Hooff JP Kootstra G In situ preservation of kidneys from non-heart-beating donors-a proposal for a standardized protocol Transplantation 1993 56 613 617 10.1097/00007890-199309000-00022 8212157

[B5] Kootstra G The asystolic, or non-heart beating, donor Transplantation 1997 63 917 921 10.1097/00007890-199704150-00001 9112339

[B6] Wheatley TJ Doughman TM Veitch PS Nicholson ML Kidney retrieval from asystolic donors using an intra-aortic balloon catheter Br J Surg 1996 83 962 963 10.1002/bjs.1800830724 8813787

[B7] Gravel MT Arenas JD Chenault R Magee JC Rudich S Maraschio M DebRoy M Miller W Punch JD Kidney transplantation from organ donors following cardiopulmonary death using extracorporeal membrane oxygenation support Ann Transplant 2004 9 57 58 15478893

[B8] Lee JH Hong SY Oh CK Hong YS Yim H Kidney transplantation from a donor following cardiac death supported with extracorporeal membrane oxygenation J Korean Med Sci 2012 27 115 119 22323856 10.3346/jkms.2012.27.2.115 PMC3271282

[B9] Gok MA Buckley PE Shenton BK Balupuri S El-Sheikh MA Robertson H Soomro N Jaques BC Manas DM Talbot D Long-term renal function in kidneys from non-heart-beating donors: A single-center experience Transplantation 2002 74 664 669 10.1097/00007890-200209150-00013 12352883

[B10] Brook NR White SA Waller JR Veitch PS Nicholson ML Non-heart beating donor kidneys with delayed graft function have superior graft survival compared with conventional heart-beating donor kidneys that develop delayed graft function Am J Transplant 2003 3 614 618 10.1034/j.1600-6143.2003.00113.x 12752318

[B11] Balupuri S Buckley P Snowden C Mustafa M Sen B Griffiths P Hannon M Manas D Kirby J Talbot D The trouble with kidneys derived from the non heart-beating donor: a single center 10-year experience Transplantation 2000 69 842 846 10755537 10.1097/00007890-200003150-00029

[B12] Daemen JW Oomen AP Janssen MA van de Schoot L van Kreel BK Heineman E Kootstra G Glutathione S-transferase as predictor of functional outcome in transplantation of machine-preserved non-heart-beating donor kidneys Transplantation 1997 63 89 93 10.1097/00007890-199701150-00017 9000667

[B13] Wilson CH Asher JF Gupta A Vijayanand D Wyrley-Birch H Stamp S Rix DA Soomro N Manas DM Jaques BC Peaston R Talbot D Comparison of HTK and hypertonic citrate to intraarterial cooling in human non-heart-beating kidney donors Transplant Proc 2007 39 351 352 10.1016/j.transproceed.2007.01.012 17362727

[B14] Wilson CH Stansby G Haswell M Cunningham AC Talbot D Evaluation of eight preservation solutions for endothelial in situ preservation Transplantation 2004 78 1008 1013 10.1097/01.TP.0000135465.00738.ED 15480166

[B15] Gok MA Shenton BK Buckley PE Peaston R Cornell C Soomro N Jaques BC Manas DM Talbot D How to improve the quality of kidneys from non-heart-beating donors: a randomized controlled trial of thrombolysis in non-heart-beating donors Transplantation 2003 76 1714 1719 10.1097/01.TP.0000093834.05766.FD 14688521

[B16] El-Mas MM Mohy El-Din MM El-Gowilly SM Sharabi FM Relative roles of endothelial relaxing factors in cyclosporine-induced impairment of cholinergic and beta-adrenergic renal vasodilations Eur J Pharmacol 2004 487 149 158 10.1016/j.ejphar.2004.01.025 15033387

[B17] Gok MA Pelsers M Glatz JF Shenton BK Peaston R Cornell C Talbot D Use of two biomarkers of renal ischemia to assess machine-perfused non-heart-beating donor kidneys Clin Chem 2003 49 172 175 10.1373/49.1.172 12507976

[B18] Livak KJ Schmittgen TD Analysis of relative gene expression data using real-time quantitative PCR and the 2(−ΔΔC(T)) method Methods 2001 25 402 408 10.1006/meth.2001.1262 11846609

[B19] Cowley RA Trump B Pathophysiology in Shock, Anoxia and Ischemia 1982 Baltimore, MD: Williams and Wilkins

[B20] Kay MD Hosgood SA Bagul A Nicholson ML Comparison of preservation solutions in an experimental model of organ cooling in kidney transplantation Br J Surg 2009 96 1215 1221 10.1002/bjs.6681 19787767

[B21] Lin Y Kirby JA Browell DA Morley AR Shenton BK Proud G Taylor RM Renal allograft rejection: expression and function of VCAM-1 on tubular epithelial cells Clin Exp Immunol 1993 92 145 151 7682162 10.1111/j.1365-2249.1993.tb05961.x PMC1554858

[B22] Bell P Dibekoglu M Gonzalez C Schlumpf R Wijnen R Results of transplantation with non-heart-beating donors Transplant Proc 1995 27 2951 2956 7482975

[B23] Bonventre JV Cheung JY Effects of metabolic acidosis on viability of cells exposed to anoxia Am J Physiol 1985 249 C149 C159 4014448 10.1152/ajpcell.1985.249.1.C149

[B24] Currin RT Gores GJ Thurman RG Lemasters JJ Protection by acidotic pH against anoxic cell killing in perfused rat liver: evidence for a pH paradox FASEB J 1991 5 207 210 2004664 10.1096/fasebj.5.2.2004664

[B25] Bronk SF Gores GJ pH-dependent nonlysosomal proteolysis contributes to lethal anoxic injury of rat hepatocytes Am J Physiol 1993 264 G744 G751 8476058 10.1152/ajpgi.1993.264.4.G744

[B26] Ahmad N Pratt JR Potts DJ Lodge JP Comparative efficacy of renal preservation solutions to limit functional impairment after warm ischemic injury Kidney Int 2006 69 884 893 10.1038/sj.ki.5000063 16407886

[B27] Ahmad N Hostert L Pratt JR Billar KJ Potts DJ Lodge JP A pathophysiologic study of the kidney tubule to optimize organ preservation solutions Kidney Int 2004 66 77 90 10.1111/j.1523-1755.2004.00709.x 15200415

[B28] Stewart ZA Lonze BE Warren DS Dagher NN Singer AL Montgomery RA Segev DL Histidine-tryptophan-ketoglutarate (HTK) is associated with reduced graft survival of deceased donor kidney transplants Am J Transplant 2009 9 1048 1054 10.1111/j.1600-6143.2008.02545.x 19298449

